# Implicit Is Not Enough: Explicitly Enforcing Anatomical Priors inside Landmark Localization Models

**DOI:** 10.3390/bioengineering11090932

**Published:** 2024-09-17

**Authors:** Simon Johannes Joham, Arnela Hadzic, Martin Urschler

**Affiliations:** 1 Institute for Medical Informatics, Statistics and Documentation, Medical University of Graz, 8036 Graz, Austria; 2 Institute of Computer Graphics and Vision, Graz University of Technology, 8010 Graz, Austria; 3 BioTechMed-Graz, 8010 Graz, Austria

**Keywords:** artificial intelligence, deep learning, digital imaging/radiology, convolutional neural network, landmark localization, a priori knowledge, anatomical constraints

## Abstract

The task of localizing distinct anatomical structures in medical image data is an essential prerequisite for several medical applications, such as treatment planning in orthodontics, bone-age estimation, or initialization of segmentation methods in automated image analysis tools. Currently, Anatomical Landmark Localization (ALL) is mainly solved by deep-learning methods, which cannot guarantee robust ALL predictions; there may always be outlier predictions that are far from their ground truth locations due to out-of-distribution inputs. However, these localization outliers are detrimental to the performance of subsequent medical applications that rely on ALL results. The current ALL literature relies heavily on implicit anatomical constraints built into the loss function and network architecture to reduce the risk of anatomically infeasible predictions. However, we argue that in medical imaging, where images are generally acquired in a controlled environment, we should use stronger explicit anatomical constraints to reduce the number of outliers as much as possible. Therefore, we propose the end-to-end trainable Global Anatomical Feasibility Filter and Analysis (GAFFA) method, which uses prior anatomical knowledge estimated from data to explicitly enforce anatomical constraints. GAFFA refines the initial localization results of a U-Net by approximately solving a Markov Random Field (MRF) with a single iteration of the sum-product algorithm in a differentiable manner. Our experiments demonstrate that GAFFA outperforms all other landmark refinement methods investigated in our framework. Moreover, we show that GAFFA is more robust to large outliers than state-of-the-art methods on the studied X-ray hand dataset. We further motivate this claim by visualizing the anatomical constraints used in GAFFA as spatial energy heatmaps, which allowed us to find an annotation error in the hand dataset not previously discussed in the literature.

## 1. Introduction

Anatomical Landmark Localization (ALL) involves the localization of distinctive points or structures in medical images, such as X-ray, MRI, or CT scans, and is a vital preprocessing step for many medical applications. For instance, locating these anatomical landmarks allows clinicians to accurately assess the severity of craniomaxillofacial malformations [1]. In addition, ALL plays an important role in treatment planning for orthodontics and orthognathic surgery using cephalometric images [2,3], assisting orthodontists in confirming diagnoses and determining retention stability and duration [4]. Automated image analysis tools also rely on anatomical landmark locations to initialize segmentation methods [5], which classify each pixel by task-specific semantic classes, e.g., finding all pixels belonging to tumors. Moreover, deformable registration methods may be built upon corresponding anatomical landmarks [6], e.g., to study lung perfusion. Other medical applications that benefit from accurate ALL prediction include the analysis of knee radiographs at different stages of osteoarthritis [7] and the guidance of visual navigation systems in orthopedic surgery [8]. However, manual ALL is error-prone, labor-intensive, time-consuming, and requires medical expertise [1]. In addition, the high variability within and between human anatomies further complicates the accuracy of manual ALL [3].

In recent years, machine-learning-based methods have been extensively explored to address ALL, as they provide the ability to approximate arbitrarily complex functions given sufficient learnable parameters and annotated training data [9,10,11,12,13]. In addition, the advent of deep learning has significantly transformed the fields of computer vision and ALL, allowing models, such as Convolutional Neural Networks (CNNs) [14], to learn task-specific features directly from data. Consequently, CNNs have been widely used to solve ALL [1,3,15,16,17]. However, even CNNs and their variants, such as the U-Net [18], struggle with the local similarity of anatomical structures and out-of-distribution inputs [15]. This limitation introduces the risk of predicting landmarks far from their true location (outlier prediction), which may hinder subsequent medical applications that rely on ALL estimates. For instance, Weng et al. [19] used anatomical landmarks to assess spinal flexibility, where an outlier could have a significant negative impact on the curvature estimate used by spine surgeons to evaluate spinal disorders. Therefore, ensuring robustness against outliers in ALL is critical to prevent potential negative medical consequences.

Several studies [1,3,15,17,20] have explored the integration of anatomical constraints into machine-learning-based approaches for ALL to enhance robustness against outliers. For instance, methods such as the SpatialConfiguration-Net (SCN) [15] and the CMF-Net [1] implicitly enforce anatomical feasibility by splitting their CNN into a localization component (providing coarse ALL estimates) and a spatial configuration component (providing anatomical refinement), assuming that the combined components are beneficial for both accuracy and robustness. Other techniques [3,17,20] integrate anatomical feasibility into the loss function, which evaluates the difference between the model output and the target. In addition, some methods [1,21,22] employ mechanisms such as attention [23] or use rich-representation learning [3,24] to help CNNs learn complex global relationships, thereby improving localization performance.

However, these methods cannot guarantee that the converged model adheres to the notion of anatomical feasibility seen during training; we argue that using the implicitly induced anatomical prior in these methods alone is not enough. The real-world clinical setting often includes out-of-distribution samples with occlusions (tags that contain medical information, field-of-view cutoffs, or missing body parts) or other variability not captured in the training dataset. Accurate ALL estimates of all visible landmarks are still required. To overcome this limitation, we propose our Global Anatomical Feasibility Filter and Analysis (GAFFA) method, which applies explicit anatomical constraints during training and inference. In addition, GAFFA allows us to visually analyze the learned anatomical constraints, as they can be represented as spatial energy heatmaps.

### Contribution

Based on the work of [15], we use a refined U-Net model for initial landmark localization, but we do not use the implicit spatial configuration component for landmark refinement in our proposed method. Instead, inspired by the works of [25,26] in the area of facial landmark localization and human pose estimation, respectively, we propose our end-to-end trainable GAFFA method that approximately solves a Markov Random Field (MRF) [27] in a fully differentiable manner. GAFFA filters out outlier predictions that are inconsistent with human anatomy, exemplified on X-ray images of hand bones. To the best of our knowledge, this has not been investigated in the ALL or medical image analysis literature. The explicit prior anatomical knowledge is provided by the topology of the MRF, which largely follows the natural bone connectivity of a human hand, and conditional distribution heatmaps, which describe the probability that landmark $l_{a}$ is in a specific location, given the location of landmark $l_{b}$. These conditional distribution heatmaps are estimated with the Gaussian Mixture Model (GMM) [28] framework, using ground-truth distances between landmarks computed with the annotated training data prior to model training. For comparison, we implemented both an SCN and an external Graphical Model (GM) [10], which also implements an MRF. However, for the GM, the separate MRF is approximately solved with Loopy Belief Propagation (LBP) [29] in a non-differentiable way. Overall, we evaluate three different landmark refinement paradigms: explicit anatomical prior (GM), implicit anatomical prior (SCN), and our proposed explicit end-to-end trainable anatomical prior (GAFFA). Our experimental results show an improvement in robustness to large outliers using GAFFA over state-of-the-art methods.

Our main contributions of GAFFA that extend [25,26] are as follows:

- We use Differentiable Spatial to Numerical Transform (DSNT) [30] coordinate regression. DSNT has no disconnect between loss computation and desired metrics and retains the spatial generalization property of heatmap regression.
- The computational costs are reduced by combining the optimization strategies of [25,26] and designing an efficient pipeline of upscaling and downscaling operations within GAFFA. This is necessary to make our method computationally feasible, as we use higher resolution inputs and a larger number of landmarks than [25,26].
- We refine the marginal energy equation used in GAFFA, e.g., returning back from logarithmic space is not necessary for finding the location of the highest pixel intensity.
- The data augmentation scheme is extended with occlusion perturbations [24], which increases the overall ALL performance and robustness against occlusions.

## 2. Methods

We follow the methodology of Payer et al. [15] to divide the task of ALL into the two subtasks of initial landmark localization and landmark refinement. Our initial landmark localization method is based on the U-Net [18], which is also used in the local appearance component of [15]. The rough localization results of the U-Net are then filtered with one of the three investigated landmark refinement methods. We implemented a SpatialConfiguration-Net (SCN), as proposed by Payer et al. [15], that exploits implicit anatomical constraints within the network architecture. Furthermore, based on the notion of Markov Random Fields (MRFs) [31], we use an external Graphical Model (GM) [10] and lastly propose the end-to-end trainable Global Anatomical Feasibility Filter and Analysis (GAFFA) method based on [25,26] to explicitly find a consensus between anatomical constraints and initial localization results. Our code and experimental setup can be found at https://github.com/imigraz/GAFFA.

### 2.1. Dataset

We used the Digital Hand Atlas dataset (https://ipilab.usc.edu/research/baaweb/dbdform/ last accessed 19 July 2024) with annotations provided at (https://github.com/christianpayer/MedicalDataAugmentationTool-HeatmapRegression/tree/master/hand_xray/hand_xray_dataset/setup last accessed 19 July 2024), which has been extensively studied in the ALL literature [15,17,20,24], where hand bone landmarks can be used as an initial stage, e.g., for bone-age estimation [32,33,34]. It contains 895 2D X-ray images of hands, each having 37 landmark annotations. There is no information about the physical resolution for this dataset, which is very important for medical applications. Hence, there is a normalization procedure found in the literature [15] that defines an image-specific normalization constant, $s^{\left(j\right)}$, which is defined as

(1) $$s^{\left(j\right)}=\frac{50}{\left|\right|l_{2}^{\left(j\right)}-l_{6}^{\left(j\right)}{\left|\right|}_{2}}\;,$$

where $l_{2}^{\left(j\right)}$ and $l_{6}^{\left(j\right)}$ denote the corresponding coordinates of Landmark 2 and Landmark 6 in the image *j*. Moreover, ${\left||.|\right|}_{2}$ describes the Euclidean distance between any two landmarks. This procedure normalizes each wrist (described by $l_{2}$ and $l_{6}$) to be 50 mm wide. We can then use $s^{\left(j\right)}$ to measure how many millimeters a pixel distance represents in image *j*.

### 2.2. Initial Landmark Localization with Heatmap Regression

**Figure 1:**
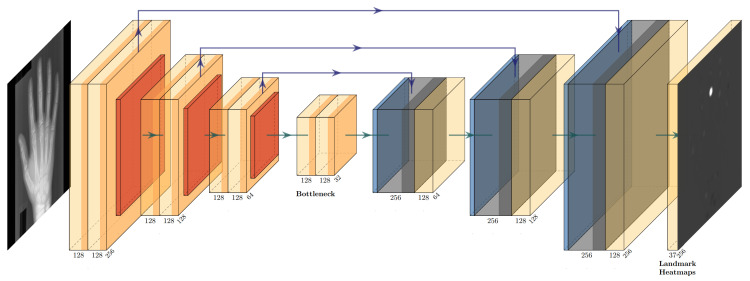
U-Net architecture used for initial landmark localization, applied to a 256×256 X-ray image of a hand from our studied dataset. We solely show one output heatmap, but there is a predicted output heatmap for each landmark. While we visualize three levels here, our implementation used four levels. Light yellow blocks indicate convolution layers in the encoder and bottleneck parts of the network, respectively, red blocks indicate average pooling layers, light blue blocks indicate bilinear upsampling, dark blue and dark yellow blocks indicate convolution layers in the decoder part of the U-Net. Purple arrows illustrate skip connections that directly connect encoder and decoder layers.

The initial landmark prediction is based on the U-Net [18] heatmap regression framework of [15], which is openly available on GitHub (https://github.com/christianpayer/MedicalDataAugmentationTool-HeatmapRegression last accessed on 19 July 2024). However, we rewrote all the TensorFlow code in this framework with PyTorch Lightning, as this allowed us to use other PyTorch-based libraries. The used U-Net, shown in Figure 1, outputs a heatmap for each unique landmark in the dataset (37 output heatmaps in total). The regression target is a Gaussian-distributed blob placed so that the blob peak coincides with the annotated target landmark location. The final coordinate prediction of a landmark can be derived by finding the location of the pixel with the highest intensity in its corresponding heatmap prediction via the non-differentiable argmax function.

The network is divided into an encoder part and a decoder part. Each part consists of four levels, and each level consists of two successive convolution layers and an upsampling or downsampling operation. Each convolution layer has 128 input channels, except for the first convolution layer in each decoder level, which expects 256 input filter maps, and the first convolution layer of the network, which processes the input grayscale image. Furthermore, each convolution layer consists of a convolution operation with a kernel size of 3×3 and stride of 1, followed by batch normalization [35], non-linear activation (LeakyReLU [36] with negative slope of 0.1), and dropout [37] (set to 0.3). Additionally, each encoder level uses average pooling with a kernel size of 2×2 and a stride of 2 for downsampling. In the decoder part of the network we used fixed bilinear upsampling, as suggested by [15]. Skip connections directly connect encoder and decoder layers, which lets the network effectively combine low and high level features. The final convolution layer of the decoder part outputs 37 heatmap predictions, one heatmap per unique landmark. The main differences to [15] are the batch normalization step after each convolution operation and two consecutive convolution layers in each encoder level instead of three.

### 2.3. Explicit Landmark Refinement with Markov Random Fields

**Figure 2:**
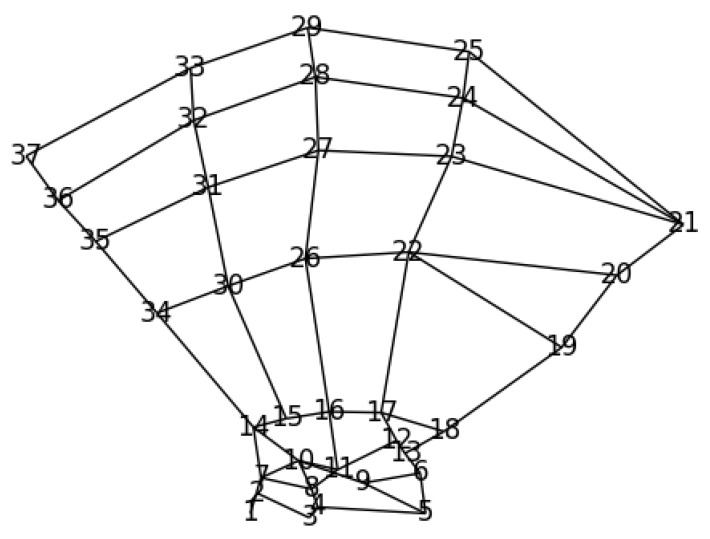
MRF topology used for this dataset. Each vertex represents one of the 37 unique landmarks in the studied hand dataset. The topology is inspired by the anatomy of the human hand, with additional edge connections between fingers to increase the spatial constraint.

The investigated explicit landmark refinement methods (GM and our proposed GAFFA) are based on the notion of Markov Random Fields (MRFs) [27,31]. An MRF is an undirected graph, *G*, with a set of vertices, v∈V, connected by edges, e∈E. Similar to [10], we let each *v* correspond to one unique landmark in our dataset and allow each *v* to have several landmark candidates. The MRF defines a discrete optimization problem, whose solution allows us to rank each landmark candidate of each *v* according to the agreement between its anatomical feasibility and the initial localization confidence of the U-Net. The definition of landmark candidates depends on the specific method. The Graphical Model has to explicitly extract landmark candidates from the U-Net heatmaps for each *v*. In contrast, our proposed GAFFA solves the MRF directly in image space and does not require explicit extraction of landmark candidates. For this method, the landmark candidates of a landmark, *v*, are all pixel locations in input image space. In addition, an MRF requires the definition of its graph topology. Differently to [10], where the topology is learned directly from the data, we manually chose a topology based on the bone connectivity observed in human anatomy, with some additional connections between fingers to further strengthen the spatial constraint, as visualized in Figure 2.

The MRF also requires the definition of unary and binary costs. The unary cost term describes the initial cost of selecting a particular landmark candidate of *v* as the final landmark location of the unique landmark represented by *v*. For unary costs, we use the pixel intensities of the respective U-Net heatmaps. The binary cost term defines the cost of choosing two landmark candidates connected by an edge, *e*, which acts as an anatomical constraint during optimization. For binary costs, both investigated methods rely on first fitting statistical distributions to the measured distances between landmarks that we can observe in the annotated training set. The fitted distributions are then used to estimate how well the observed distances between predicted landmark candidates match our prior anatomical knowledge. Finding the exact solution of the MRF is NP-hard in a cyclic graph [38]. Hence, we approximate the global solution to this optimization problem in both methods with implementations of the sum-product algorithm [39].

### 2.4. End-to-End Trainable Global Anatomical Feasibility Filter and Analysis

GAFFA is inspired by the works of [25,26]. In their approaches, $p(x_{i})$ is defined as the unary marginal distribution representing the probability that landmark $l_{i}$ is located at the random variable $x_{i}=\left(u_{i},v_{i}\right)\in R^{2}$, which represents a 2D image location. For simplicity, $p_{i}$ denotes $p(x_{i})$, and $\hat{p_{i}}$ denotes the approximation of $p_{i}$ provided by GAFFA. We adopt their definition of the unary marginal probability of $l_{i}$ computed over all landmarks in its extended neighborhood, $N_{i}\subset V$, in our defined MRF topology, expressed as

(2) $$\hat{p_{i}}=\frac{1}{Z}\prod_{j\in N_{i}}p_{i|j}*p_{j}+b_{j\to i}\;,$$

where $b_{j\to i}$ is the bias describing the probability of a message passing from $l_{j}$ to $l_{i}$, *Z* is a normalization constant (which is discarded in the implementation of [25,26] as they treat the distributions as energies, focusing only on the maximum value in the final heatmaps), $p_{i|j}$ is the conditional probability of $l_{i}$ given $l_{j}$ estimated prior to model training, and * denotes the convolution operator. According to [26], this equation represents an iteration of the sum-product algorithm, which we also use to solve the MRF described by the GM.

Following [25], we define $N_{i}=LN_{i}\cup GN$ as the combination of the local neighborhood $LN_{i}\subset V$ of $l_{i}$ and a fixed set of global landmarks, GN⊂V, that do not need to be directly connected to $l_{i}$ in the MRF topology. $LN_{i}$ contains only the nearest neighbors, which are all landmarks connected to $l_{i}$ by an edge, *e*. For GN, we manually include $l_{2}$ and $l_{6}$, located on the wrist, as illustrated in Figure 2. This choice is based on the fact that the wrist is the least likely region to be occluded in clinical settings. We also include {$l_{14},\cdots ,l_{18}$}, which mark the metacarpophalangeal joints, and {$l_{21},l_{25},l_{29},l_{33},l_{37}$}, which mark the fingertips, to represent the global structure of a human hand. The inclusion of GN in $N_{i}$ is crucial for robustness against occlusion. In extreme cases, where the entire local neighborhood of $l_{i}$ is occluded, the position can still be roughly estimated using the global landmarks.

#### 2.4.1. Prior Anatomical Knowledge and GAFFA

Unlike the SCN, which only incorporates implicit anatomical constraints into its network architecture, GAFFA uses explicit anatomical priors. We followed a method similar to [25] to estimate each $p_{i|j}$, which serve as anatomical priors that can be further optimized during training as they are used to initialize convolution kernels. However, the training images and their corresponding ground truth coordinates are augmented with the procedures explained in Section 3.2. Therefore, for a more robust estimate of $p_{i|j}$, we already sample each training image three times to account for multiple spatial transformations for each landmark in the prior before starting model training. For each training image, we first translate landmark $l_{i}$ using the translation vector

(3) $$T_{i|j}=\mathrm{center}\;\mathrm{of}\;\mathrm{frame}-\left(u_{j},v_{j}\right)\;,$$

which moves landmark $l_{j}$ to the center of the image. We then use the Gaussian Mixture Model (GMM) framework [28] to estimate each $p_{i|j}$ combination. The GMM approach fits a mixture of several Gaussian distributions to the data. Through empirical experimentation with up to 10 different Gaussian distributions, we determined the optimal number by selecting the one with the lowest mean between Akaike Information Criterion (AIC) and Bayesian Information Criterion (BIC) values.

**Figure 3:**
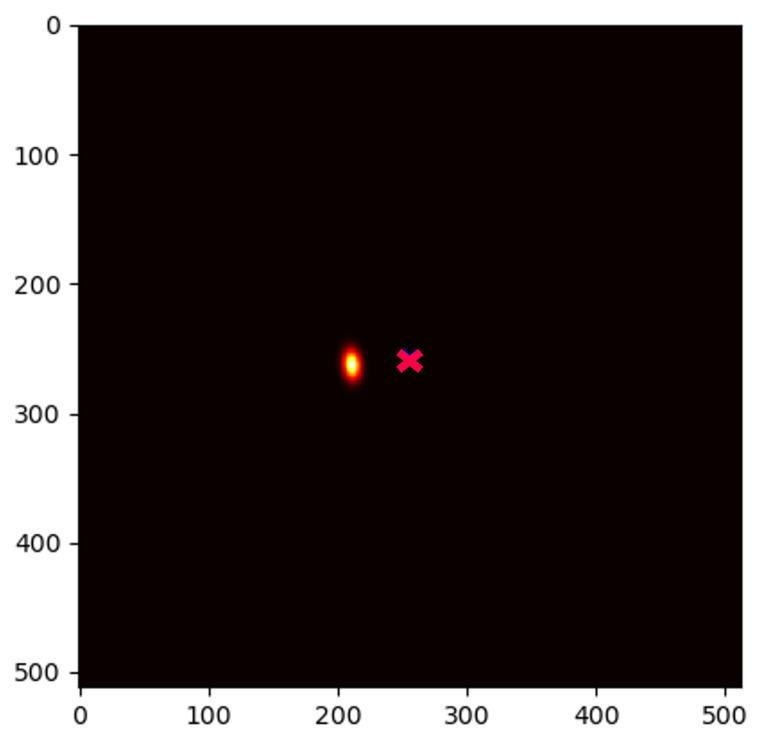
Conditional distribution heatmap $p_{2|6}$, learned from 597 unique images of the investigated hand dataset, where the red cross marks the center of the frame (representing the location of $l_{6}$) and the heatmap indicates where $l_{2}$ is likely to be located relatively to $l_{6}$.

Due to the application of $T_{i|j}$, there may be responses outside the initial image boundary set by the input images. Therefore, the resulting $p_{i|j}$ heatmaps have twice the resolution of their input images. Consequently, Figure 3 shows a 512×512 image illustrating the GMM result of $p_{l_{2}|l_{6}}$, even though the original input images it was trained on had an image resolution of 256×256. Also, Figure 3 shows where Landmark 2 should be, given the location of Landmark 6, which was translated to the center of the image.

#### 2.4.2. Numerical Realization

We implement a modified Equation (2) from [25] that no longer quantifies a probability for $\hat{p_{i}}$, but an energy:

(4) $$\hat{me_{i}}=me_{i}+\sum_{j\in N_{i}}log\left[Softplus_{\beta }\left(p_{i|j}\right)*Softplus_{\beta }\left(p_{j}\right)+Softplus_{\beta }\left(b_{j\to i}\right)+\epsilon \right]\;,$$

where $\hat{me_{i}}$ is the predicted energy heatmap of landmark $l_{i}$ and $me_{i}=log\left[Softplus_{\beta }\left(p_{i}+\epsilon \right)\right]$ and $Softplus_{\beta }\left(x\right)=\frac{1}{\beta }\log\left(1+e^{\beta x}\right)$ with β=5 and $\epsilon =10^{-6}$ in all experiments. The product operation in Equation (2) is replaced by a summation of logarithmic terms to improve numerical stability. In addition, the Softplus function ensures a positive convolution output, thereby mitigating numerical instability, as noted by [26]. Unlike [25,26], we do not perform a final conversion from logarithmic space, since our focus is on the maximum value, which remains unchanged when not converted from logarithmic space. Additionally, we apply Softplus to the bias, similar to [26].

The convolution operation described in Equation (4) is computationally intensive because it requires $p_{i|j}$ to be twice the size of $p_{j}$ and uses $p_{i|j}$ as a learnable convolution kernel. To make Equation (4) computationally feasible, we use both the grouped convolution strategy from [25] and the Fourier space convolution method from [26]. Our implementation uses a Fast Fourier Transform (FFT) in PyTorch (https://github.com/fkodom/fft-conv-pytorch last accessed: 7 June 2024), which supports group convolutions. In addition, we heavily downsample $p_{i|j}$, $p_{j}$, and $b_{j\to i}$ to minimize the number of learnable parameters and reduce computational time of the sum in Equation (4). This downsampling is similar to the spatial component reduction in the SCN, but we use bicubic interpolation instead of average pooling to preserve the shape of the original conditional distribution and localization heatmaps. However, the original heatmap resolution is retained for $me_{i}$ to preserve the initial accuracy of the localization model. Consequently, the final energy sum must be upsampled using bicubic interpolation to match the heatmap resolution of the localization model for the final addition with $me_{i}$. Finally, due to the need to manually initialize spatially variant kernel weights, we invert our previously estimated kernels along their spatial dimensions to accommodate PyTorch’s use of cross-correlation rather than convolution (https://pytorch.org/docs/stable/generated/torch.nn.Conv2d.html last accessed 7 June 2024), thereby ensuring the correct spatial orientation of the conditional heatmaps.

Overall, we configured GAFFA to regress coordinates following the methodology proposed by [25]. However, the coordinate regression technique is not elaborated in detail by [25]. Consequently, we have chosen the Differentiable Spatial to Numerical Transform (DSNT) approach [30] without regularization. This choice allows us to avoid enforcing a strict target heatmap with a single Gaussian blob for each landmark. The DSNT approach is fully differentiable, ensuring that there is no disconnect between the loss function and the metric of interest. In addition, we use the Mean Squared Error (MSE) loss to compute the discrepancy between predicted and target coordinates, as opposed to the Euclidean distance loss proposed by [30]. This decision is based on the observation that the Euclidean distance loss requires an additional square root computation, while the optimization objective remains essentially the same.

#### 2.4.3. GAFFA Landmark Refinement Flowchart

**Figure 4:**
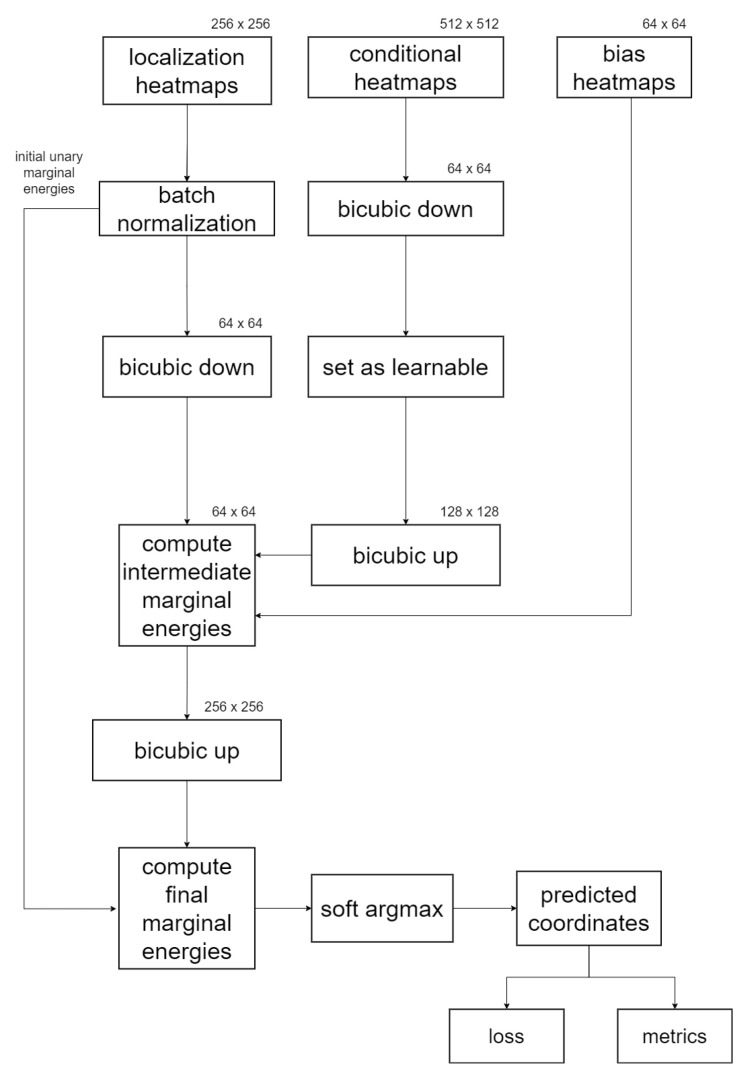
Flowchart of GAFFA when applied to 256×256 output heatmaps of the U-Net.

Figure 4 presents an abstract flowchart illustrating the coordinate regression process of GAFFA, assuming a 256×256 image resolution for our localization model. Initially, we apply batch normalization to the U-Net localization heatmaps to expedite convergence, as suggested in (https://github.com/max-andr/joint-cnn-mrf last accessed: 7 June 2024), which implements [26]. We then perform bicubic downsampling on the batch-normalized heatmaps and the conditional distribution heatmaps. We keep the conditional heatmaps at a much lower resolution than required for the convolution in Equation (4) to minimize the number of trainable parameters. This requires an upsampling step to restore the conditional heatmaps to twice the resolution of the localization heatmaps. Furthermore, we keep the localization heatmaps at their original resolution in memory for the computation of $me_{i}$, as indicated by the line connecting the batch normalization phase to the final $\hat{me_{i}}$ computation. The bias terms $b_{j\to i}$ are interpreted as heatmaps, with each pixel initialized with ϵ. After computing the sum in Equation (4), we upsample the intermediate results back to the original resolution before integrating them with $me_{i}$, resulting in $\hat{me_{i}}$. Finally, the refined landmark coordinates are extracted from each $\hat{me_{i}}$ using the soft argmax function adopted from [30], and these are subsequently used for loss and performance evaluation.

#### 2.4.4. Comparison with SCN and Graphical Model

Our proposed method essentially combines the ideas of the SCN and the GM. It follows the idea of the SCN having subsequent end-to-end trainable components within the network for landmark localization and refinement. However, the landmark refinement component of our method approximately solves an MRF, similar to the GM, which explicitly enforces anatomical constraints. In addition, unlike the GM, the anatomical prior is further refined during training.

**External Graphical Model.** The Graphical Model (GM) is directly inspired by [10] and requires the explicit extraction of landmark candidates for every *v* from the U-Net prediction heatmaps. For this, we used a blob extraction method (Laplacian of Gaussian) that returns the location of blob peaks, as we assume Gaussian-distributed blobs near target locations. We rank all found candidates of a heatmap based on their blob peak intensity, $b_{i}$ (larger is better), which also defines the unary cost of choosing the corresponding landmark candidate. We further process only the top 25 candidates based on this ranking to reduce computation time on the computationally expensive binary cost estimation. Moreover, in all experiments we ignore normalized blob peak values, $n_{i}$, below the threshold of 0.015, with $n_{i}$ defined as

(5) $$n_{i}=\frac{b_{i}}{\sum_{i=1}^{N}b_{i}},$$

where *N* is the number of landmark candidates of one landmark.

We combine two types of binary cost terms that are weighted to be equally important. For each edge, *e*, we use ground-truth distances to model a t-distribution of distances between landmarks connected to *e*. Furthermore, for each *e*, we perform ratio comparisons between the estimated $\mu_{e}$ of the t-distributions and the measured distances between pairs of landmark candidates with landmark pairs of a fixed set of edges that have the three lowest estimated $\sigma_{e}$ of all t-distributions. The motivation behind these ratio terms is the fact that this dataset has no physical resolution, and ratio estimates are invariant to the required distance normalization, which may be inaccurate in inference since it depends on the accurate localization of two landmarks. After cost estimation, the MRF is approximately solved in a non-differentiable way using Loopy Belief Propagation (LBP) [29], which is a variant of the sum-product algorithm. The solver (https://github.com/mbforbes/py-factorgraph last accessed on 22 July 2024) we use is based on factor graphs [39].

**Implicit Landmark Refinement with SCN.** The SCN of Payer et al. [15] uses a U-Net model for initial landmark localization, which is expected to produce localization results with high local accuracy but requires global disambiguation after convergence. The spatial configuration component then further processes the heavily downsampled U-Net output with multiple successive convolution layers without further internal downsampling and is expected to converge to a state where it outputs a spatial filter heatmap that can be used for landmark refinement of the initial U-Net outputs.

For comparison, we re-implemented the SCN within our framework. For the localization component, we used the U-Net already described in the previous section. Our spatial configuration component consists of three successive convolution layers with a kernel size of 7×7 (Payer et al. use 11×11 kernels, because they train on 512×512 input images) and 128 feature maps to take advantage of a large receptive field for landmark disambiguation. Compared to the original SCN, we also added a batch normalization layer before each activation function.

### 2.5. Rich-Representation Learning with Occlusions

**Figure 5:**
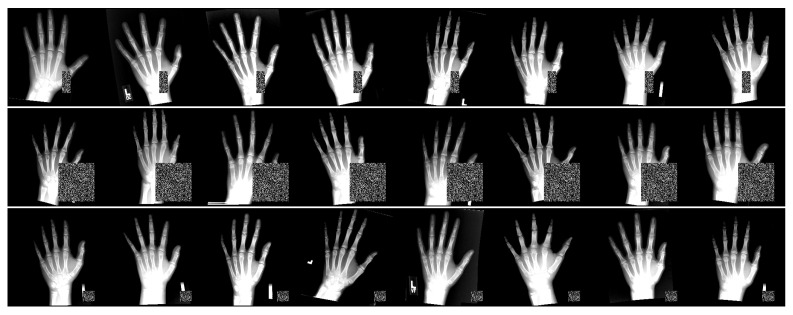
Examples of perturbed images from 3 batches of 8 input images used in training. Each image in a batch has the same perturbation. Bright regions are regions with intensities above 1.0 (due to data augmentation) and are clamped to 1.0 for visualization purposes (stronger contrast between artificial occlusions and bone tissue).

Inspired by [3,24], we incorporate occlusion perturbations into our data augmentation process. Figure 5 shows examples of the perturbed images used during training. These images underwent additional augmentation, as described in Section 3.2, before the perturbations were applied. Each batch of images is subjected to identical perturbations, potentially occluding different landmarks depending on the spatial transformation applied prior to perturbation. The occlusion boxes are randomly sized to cover between 1 and 15% of the pixels of each input image in our experiments. In contrast to [24], we focus exclusively on the full occlusion strategy, which involves boxes that completely eliminate local appearance information from parts of the input images before they are processed by the localization model. This approach is clinically relevant to our dataset, as it simulates scenarios such as missing fingers or field-of-view cut-offs. In addition, this method is more general, simulating occlusion in different regions of the hand or wrist.

To prevent the model from overfitting to the centers or intensities of the occlusion boxes, we randomize the pixel intensities and vary the size and placement of the boxes within the scene, potentially avoiding any part of the hand or wrist. Consequently, this perturbation strategy can completely obscure the local information of multiple landmarks in an image, depending on the randomized position of the box. This differs from the protocol in [24], which used uniform black and white boxes with positions randomized close to the ground truth landmark location. Our approach forces the models to rely on global features to make accurate predictions for the occluded landmarks, since the target landmarks used for loss computation remain unchanged. It also improves the overall robustness to noisy structures that are not part of the hand or wrist. Therefore, it applies an implicit anatomical constraint during training.

## 3. Experimental Setup

Our evaluation procedure consists of two test settings. We adopt the *standard test setting* that uses three-fold cross validation with the same cross-validation split that can be found in the literature [15,17]. Thus, each fold contains 597 training images and 298 test images, and for all experiments we use the average test performance over all folds, except for the number of outliers, which we sum over all folds. Furthermore, we propose a new *occlusion test setting* that extends the cross validation of the *standard test setting* by introducing artificial occlusions to the test images.

### 3.1. Evaluation Metrics

We use the evaluation metrics described in [15], which are commonly used in the ALL literature. The Point-to-Point Error (PE) describes the general accuracy measurement of ALL approaches and depends on the previously described normalization constant $s^{\left(j\right)}$. Therefore, the PE between a predicted landmark position, $p_{i}^{\left(j\right)}$, of landmark $l_{i}$ and the target position, $t_{i}^{\left(j\right)}$, in image *j* is defined as

(6) $$\begin{array}{c} PE_{i}^{\left(j\right)}=s^{\left(j\right)}\left|\right|p_{i}^{\left(j\right)}-t_{i}^{\left(j\right)}{\left|\right|}_{2}\;. \end{array}$$

Additionally, we compute the arithmetic mean (μ), Median (MD), and Standard Deviation (SD) of the PE for all landmarks in image *j*. Furthermore, the PE for all images and landmarks is denoted as $PE_{all}$. Since robustness against outlier predictions is particularly important for ALL, the literature also suggests evaluating the number of outliers exceeding a certain radius, *r*, from the target location ($\#O_{r}$) and their total percentage ($\%O_{r}$). Specifically, we evaluate outliers that are 2 mm, 4 mm, or 10 mm away from their target location (*r* > 2 mm, *r* > 4 mm, *r* > 10 mm).

### 3.2. Preprocessing and Data Augmentation

We adopt the preprocessing and data augmentation steps from (https://github.com/christianpayer/MedicalDataAugmentationTool-HeatmapRegression/blob/master/hand_xray/dataset.py last accessed: 25 July 2024), implemented using the SimpleITK library (https://simpleitk.org/ last accessed: 25 July 2024). Our approach uses on-the-fly data augmentation, starting with reducing each image to a resolution of 256×256. Spatial transformations are then applied using linear resampling, including translation, rotation, and elastic deformation. For translation, images are randomly shifted by [−10, 10] pixels, and rotations are randomly applied within [−0.2, 0.2] radians, both sampled from a uniform distribution. For elastic deformations, points on a 5×5 grid are randomly shifted by 20 pixels, followed by a third-order B-spline interpolation.

Following spatial transformations, we perform intensity-based data augmentation. Initially, pixel values are normalized between [−1, 1]. A shift operation is then applied, adding a random value within [−0.15, 0.15] to each pixel. Subsequently, each pixel, *p*, is updated using a value, *v*, sampled from the same interval, according to the equation:

(7) $$v_{new}=v_{old}\left(1+v\right)\;,$$

where $v_{new}$ is the new pixel value after scaling and $v_{old}$ is the original pixel value.

For each image, we generate 37 target heatmaps, each corresponding to a unique landmark. These heatmaps all have pixels set to 0, except pixels near the target location, where a Gaussian-distributed blob with a default σ of 3 pixels is placed. Additionally, a scaling factor of γ=1000 is applied to each pixel of the target heatmaps to ensure numerical stability, as no significant loss improvement was observed during training without γ, as noted by [15]. These heatmaps undergo the same spatial transformations (translation, rotation, and elastic deformation) as the input images.

### 3.3. Model Training

Dataset-specific hyperparameters are directly used from [15], e.g., σ of 3 pixels for the Gaussian blobs used for generating target heatmaps. We evaluated the performance of each model after training for a fixed number of 800 epochs, using a batch size of 8 for all experiments. During each epoch, the model processes the entire training dataset once. To ensure a fair comparison, we did not use early stopping. Additionally, we exclude the computation of landmark predictions rotated out of the image after data augmentation and preprocessing from the loss. As a result, we observed slower but more stable convergence because not all predicted landmarks contribute to each gradient update, justifying the increased number of epochs, compared to [15], which used only 50 epochs. Moreover, we employed the ADAM optimizer [40] instead of the stochastic gradient descent algorithm used in [15], with an initial learning rate of $10^{-3}$ to facilitate faster convergence and achieve good performance without extensive hyperparameter tuning. To account for the difference in magnitude between the losses of U-Net and GAFFA, we scale them to have the same magnitude in the first batch of the first epoch. Furthermore, we train the U-Net and GAFFA end-to-end in a single training run, differently to [25,26].

### 3.4. Occlusion Test Setting Details

To the best of our knowledge, existing methods in the literature evaluate the ground truth landmark locations in the test set without introducing artificial complexity. Given that state-of-the-art models [20] nearly achieve the required clinical performance with all detected landmarks within 2 mm of the target [3], we propose a more complex test scenario that incorporates occlusions. The motivation for including artificial occlusions is to better simulate clinical conditions where patients may have missing fingers or tags may occlude parts of the hand. In addition, misaligned fields of view or imaging artifacts may introduce additional occlusions. In such cases, ALL methods should still accurately predict non-occluded landmarks and, if necessary, estimate the likely location of missing or occluded landmarks.

Since we treat all landmark predictions equally in the evaluation, we actually test the model’s understanding of global relationships between image features. When a finger is occluded, a human can still vaguely predict the position of each joint based on the appearance of neighboring fingers. Similarly, models need to understand human anatomy in order to make meaningful predictions. Thus, our proposed *occlusion test setting* allows us to evaluate the ability of CNN architectures to learn complex global relationships between image features.

The first step in our occlusion testing pipeline is to group all five landmarks belonging to a unique finger, denoted as $F_{a}=\left\{l_{v},\ldots ,l_{w}\right\}$, where a={1,…,5}. The exception is the thumb, which is represented by only four landmarks. Thus, we have a total of 24 different landmarks representing all the finger joints that can be occluded in our *occlusion test setting*. For each image *j* that we want to test, we first uniformly sample random fingers, where each finger has a $\frac{1}{5}$ chance of being selected for further occlusion testing. Next, we uniformly sample pairs of landmarks ($l_{i}$ and $l_{i+1}$) to determine which should be occluded for each selected finger. We also allow for no landmarks to be occluded in the sampled fingers. Therefore, there is still a small chance that no occlusion will be introduced in a particular test image.

The second step of our test scenario takes the randomly selected landmark pairs and occludes the region between them to simulate a missing part of the finger, potentially removing the local appearance information of several whole fingers. The occlusion of a landmark, $l_{i+1}$, is simulated by drawing multiple circles with a fixed radius, *r* (set to 10 for 256×256 images), between the neighboring landmarks $l_{i}$ and $l_{i+1}$. For this, we compute the vector $v_{i+1}$ between the 2D coordinates of $l_{i}$ and $l_{i+1}$ with

(8) $$v_{i+1}=\left(\begin{array}{c} l_{i}^{\left(x\right)}-l_{i+1}^{\left(x\right)}\\ l_{i}^{\left(y\right)}-l_{i+1}^{\left(y\right)} \end{array}\right)\;,$$

where $l_{i}^{\left(x\right)}$, $l_{i+1}^{\left(x\right)}$ and $l_{i}^{\left(y\right)}$, $l_{i+1}^{\left(y\right)}$ mark the *x* and *y* components of the landmarks $l_{i}$, $l_{i+1}$, respectively.

In addition, we calculate the number of circles, *c*, to draw between $l_{i}$ and $l_{i+1}$ with

(9) $$c=\left\lceil \frac{∥v_{i+1}∥}{r}\right\rceil \;.$$

We also use *c* and $v_{i+1}$ to define vector $t_{i+1}$ with

(10) $$t_{i+1}=v_{i+1}\frac{1.0}{c}\;,$$

where $t_{i+1}$ lies in the span of $v_{i+1}$ and can be used to calculate the position of the first circle center when added to the coordinates of $l_{i+1}$. To calculate the remaining circle centers, we scale $t_{i+1}$ by scalar *k*, which is the index of the circle we are interested in, before adding it to the point location of $l_{i+1}$. Finally, we fill all circles that are defined with their center location and radius, *r*, with the intensity value of zero and apply Gaussian noise (μ=0 and σ=0.02) to make the occlusion intensities vary.

**Figure 6:**
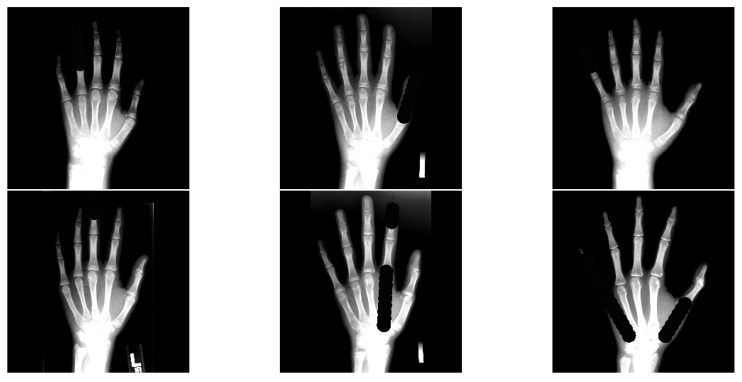
Six example test images with artificial finger occlusions. Bright regions are regions with intensities above 1.0 (due to data augmentation) and are clamped to 1.0 for visualization purposes (stronger contrast between artificial occlusions and bone tissue).

Figure 6 shows example results of our occlusion algorithm. We allow a random mixture of partial and full finger occlusions approximated by circles. Due to the fact that these occlusions are random, there may be sampled occlusion test sets with different levels of difficulty. Therefore, when performing experiments in the *occlusion test setting*, we average the performance over 30 randomly sampled test runs per cross validation fold to take advantage of the law of large numbers to make the performance evaluation account for the introduced randomness. This also makes it harder for models to overfit on the test setting.

## 4. Results

This section reports the experimental results, focusing on the evaluation of landmark refinement methods. First, we primarily evaluated the impact of rich-representation learning with occlusions on the U-Net, as it is the backbone that precedes all landmark refinement methods. Due to the general performance improvement of this perturbation strategy, we kept rich-representation learning enabled for most of the subsequent experiments.

We evaluated the performance of the investigated landmark refinement methods on the discussed *standard test setting* and *occlusion test setting*. Furthermore, we compare our best performing GAFFA model with state-of-the-art methods from the literature on the *standard test setting*. Finally, we show visual results of GAFFA, highlighting its ability to intuitively represent the enforced anatomical constraints as spatial energy heatmaps.

In terms of average inference times, one image took 0.12 s for U-Net, 0.12 s for SCN, 1.81 s for U-Net+GM, and 0.66 s for U-Net+GAFFA. We made these performance measurements on a machine with a GTX 1060 GPU and Intel i5 6600K CPU by averaging over 90 test folds computed in the *occlusion test setting*.

### 4.1. Rich-Representation Learning with Occlusions

Exposing the models to occlusions during training largely improved performance across the board, except for the median (MD) $PE_{all}$ metric, as can be seen in Table 1. The initial localization performance of the SCN improved the most when occlusion perturbations were introduced. Inspired by [3], we also investigated the effect of Laplacian blobs, as this distribution gives more importance to the close proximity of the blob peak location. We observed that adding artificial occlusions in training also improves performance in this setup. In addition, we saw slightly better performance when training the U-Net with Laplacian blobs in the *standard test setting*, even without rich-representation learning. Furthermore, Table 1 also shows that models trained with different blob distributions perform similarly when artificial occlusions are used in training. Except for the reported number of outliers above 4 mm, the U-Net trained with Laplacian blobs has slightly fewer outliers in this scenario. Due to the overall improvement in performance, we continued to use rich-representation learning implemented with artificial occlusions in subsequent experiments.

### 4.2. Landmark Refinement Methods and Standard Test Setting

In the *standard test setting*, the initial localization results of the U-Net trained with rich-representation learning are hard to outperform with our investigated landmark refinement methods, as depicted in Table 2. Post-processing with the Graphical Model severely degrades performance in most metrics, besides a standard deviation of $PE_{all}$ and a number of large outliers (r > 10 mm). The SCN reports a higher amount of small outliers (r > 2 mm) than using the U-Net alone, but improves in filtering larger outliers (r > 4 mm). Training a U-Net with GAFFA, but only evaluating the U-Net component (**U-Net**+GAFFA), leads to a similar performance as training the U-Net alone. However, the GAFFA model that was trained together with the U-Net from scratch in an end-to-end manner shows higher robustness against large outliers and slightly smaller SD. This result comes at the cost of a slightly increased mean and median of $PE_{all}$. For completeness, we also trained a U-Net with GAFFA using target heatmaps with Laplacian distributed blobs, which showed similar performance to using Gaussian-distributed blobs in this test setting.

### 4.3. Landmark Refinement Methods and Occlusion Test Setting

The proposed *occlusion test setting* is much more challenging than the *standard test setting* used in the literature, as can be seen by comparing the reported performance in Table 2 to Table 3. This test setting further highlights the importance of rich-representation learning in ALL, because models that have been trained Without Artificial Occlusions (WO) perform very poorly in this test setting. This is true for both the U-Net and the SCN models. Surprisingly, the U-Net, with rich-representation learning enabled, largely outperforms the SCN in most metrics in the *occlusion test setting*. The Graphical Model improves robustness to large outliers and greatly reduces the SD of $PE_{all}$ compared to using the converged U-Net alone, at the cost of larger median and mean $PE_{all}$, and a larger number of small outliers. In this test setting, we also observed the best overall performance by using the output of GAFFA trained end-to-end with a U-Net from scratch. GAFFA seems to be a good compromise between the high robustness against large outliers of the Graphical Model and the high local accuracy of using the U-Net alone. For completeness, we also trained this model combination with Laplacian blobs, which resulted in even better performance in this test setting. Finally, we can observe in Table 3 that U-Net models evaluated, without their end-to-end trained GAFFA component, show a much lower SD than training and using the U-Net model alone.

### 4.4. Comparison to State-of-the-Art

In Table 4, we summarize several reported results using the *standard test setting* of state-of-the-art methods and compare them to our best model. It is evident that the methods in the literature typically train on higher-resolution images. While higher resolution generally leads to higher accuracy, as indicated by lower mean and median $PE_{all}$ values for models trained on more input pixels, we argue that high robustness to large outliers does not require a high resolution.

Our model outperforms the baseline model from Payer et al. [15] across all metrics, except for the mean and median $PE_{all}$, despite being trained on only a quarter of the pixels. In addition, we outperform a recent method [17] across all metrics, which also incorporates a strong spatial prior during training. Both methods have public code showing that they use the same cross-validation split.

However, our method is outperformed by the approaches introduced in [20,24], in all metrics except for the number of outliers more than 10 mm from the target. To the best of our knowledge, no other method in the literature matches our robustness to large outliers in this hand dataset, except for the results reported by Viriyasaranon et al. [22]. However, the published code on their linked GitHub repository (https://github.com/seriee/Multiresolution-HTC last accessed 27 July 2024) lacks ALL-related code, and key linked submodules are inaccessible. In addition, they did not specify whether or not they followed the same cross-validation protocol provided by [15], and there is no information on the dataset splits used, which prevents us from reproducing their results. Therefore, we only include their results for completeness. Additionally, we have taken the performance results for [3] from [20], as the original paper does not discuss the performance of their model on the studied hand dataset.

### 4.5. Qualitative Results of GAFFA and Outlier Analysis

**Figure 7:**
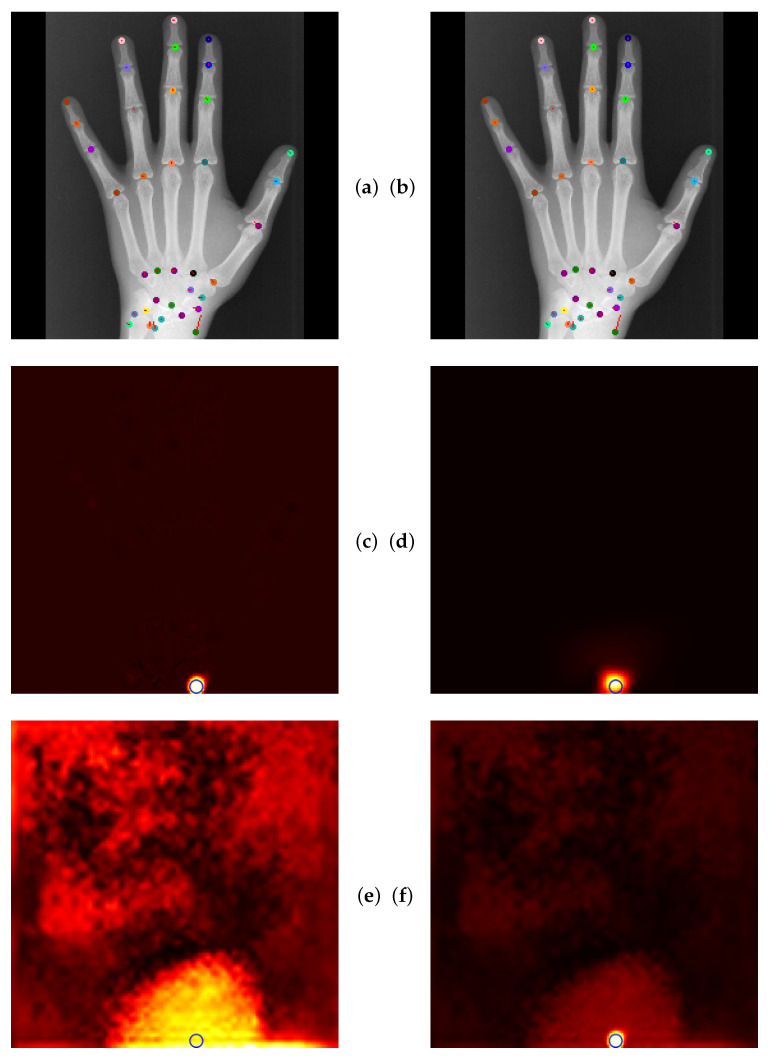
(**a**) U-Net localization output with outlier prediction of landmark $l_{5}$. (**b**) GAFFA output, the large outlier remains. (**c**) Initial marginal energy of $l_{5}$ shown as heatmap, i.e., brighter pixels have more energy. Blue circle marks the final GAFFA prediction. (**d**) Marginal energy post sum-product iteration (no bias energy). (**e**) Bias energy heatmap of $l_{5}$. (**f**) Final marginal energy. GAFFA largely agrees with the initial U-Net prediction. However, the predicted landmark location $l_{5}$ is more than a centimeter away from its annotation. This strongly suggests that the annotation of $l_{5}$ is not correctly placed, as it violates the global anatomical feasibility constraint enforced by GAFFA.

**Figure 8:**
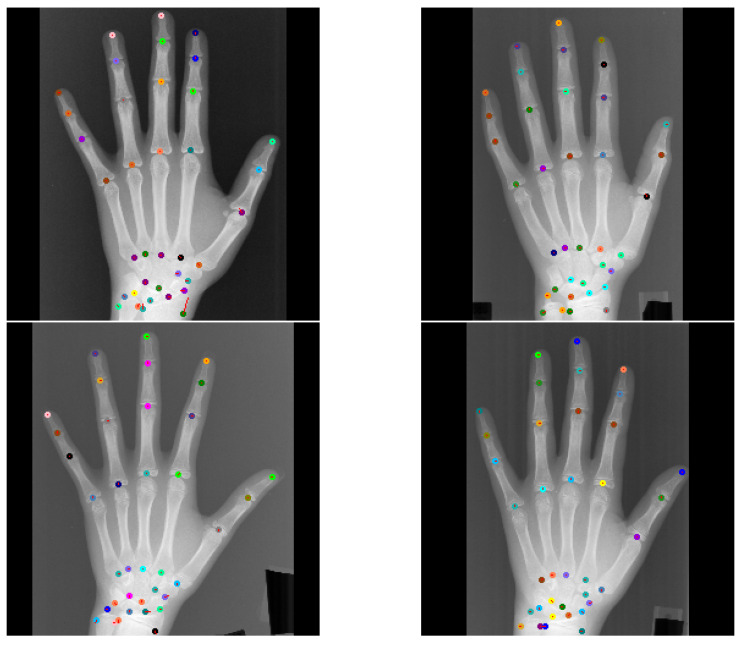
Top left image shows visual GAFFA output with the only reported large outlier (r > 10 mm) in the *standard test setting* due to an annotation error. Other images show that similar predictions of the same landmark $l_{5}$ were accurate. This indicates that the annotation of $l_{5}$ is not placed correctly in the top left image. Colored circles mark predicted landmark positions and red lines show the distance between predicted location and target location.

Global Anatomical Feasibility Filter and Analysis (GAFFA) makes it possible to illustrate the learned anatomical constraints in an intuitive way by visualizing the marginal energies of Equation (4) as heatmaps. In the case of the remaining large outlier that we reported in Table 4, we can see that the U-Net and the explicitly enforced anatomical constraint within GAFFA largely agree on the same location, as shown in Figure 7. Hence, we suspect that the outlier prediction is due to an annotation error. In addition, we performed a visual comparison with other input images and their annotations, which also supports this claim, as shown in Figure 8. Therefore, there are strong arguments for considering the position of landmark $l_{33}$ of image 3836 as an annotation error in this dataset, which, to the best of our knowledge, has not been discussed in the literature.

**Figure 9:**
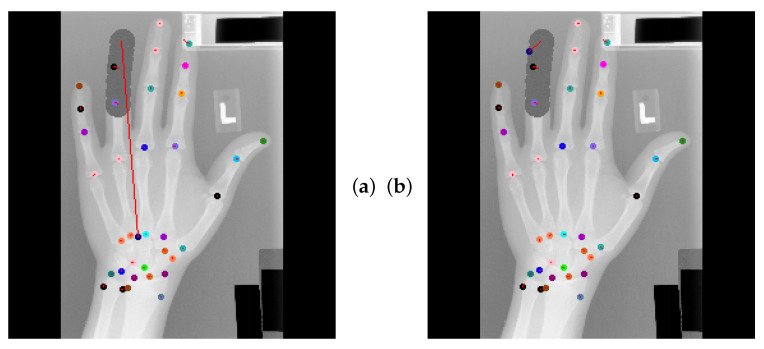

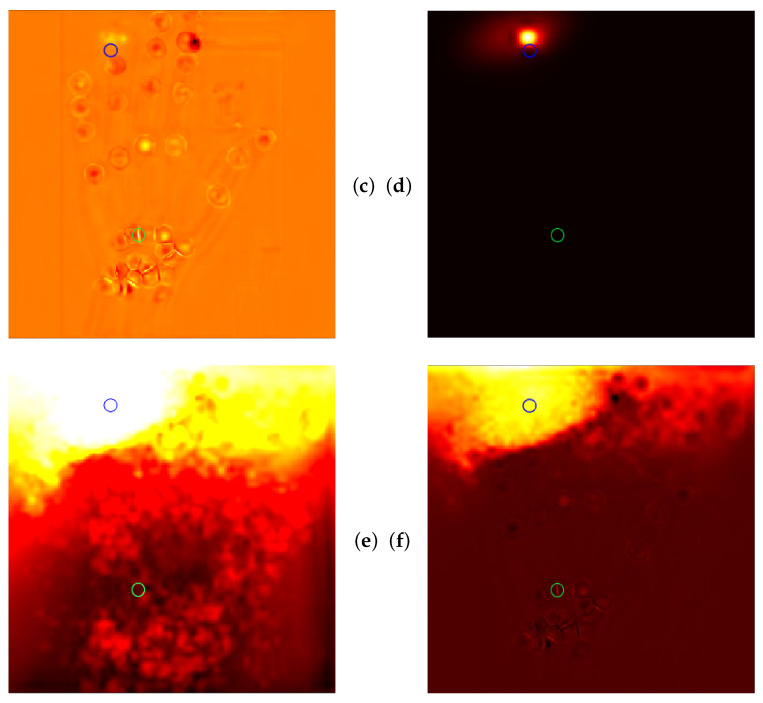
(**a**) U-Net localization output with huge outlier prediction (red line) of landmark $l_{33}$ that is artificially occluded. (**b**) GAFFA output pulling outlier closer to target. (**c**) Initial marginal energy of $l_{33}$. Green circle marks the U-Net prediction. Blue circle marks the final GAFFA prediction. (**d**) Marginal energy post sum-product iteration (no bias energy). (**e**) Bias energy heatmap of $l_{33}$. (**f**) Final marginal energy. This example shows that implicit anatomical constraints can improve overall ALL performance and make models understand the global relationships between anatomical structures. However, the explicit anatomical constraints enforced by GAFFA are needed to further reduce the risk of outlier predictions.

We can also see in Figure 9 that the U-Net trained with rich-representation learning still benefits from the introduction of GAFFA. One of the initial U-Net predictions in this example is several centimeters away from the target location, which GAFFA can pull much closer to the annotated target location. Furthermore, this figure shows that the U-Net alone, when trained with artificial occlusions, can understand global relationships between anatomical features and thus accurately localize occluded landmarks where local appearance information is no longer present. However, it cannot be expected that the exact position of landmarks near the fingertips is found based on global information alone, since these landmarks exhibit a high degree of local spatial variety.

## 5. Discussion

This section begins with a short discussion of rich-representation learning with occlusions, which improved the performance of our localization model across all metrics. This is followed by a detailed examination of each landmark refinement method.

### 5.1. Rich-Representation Learning with Occlusions

Our experiments show that incorporating rich-representation learning enables the U-Net to implicitly learn global relations between anatomical structures, consistent with the findings in [3,24]. Our results indicate that the attention mechanism is not required for ALL models to effectively learn global features, contrary to the belief in [1,21]. Consequently, the performance of all our models improved in all scenarios when occlusion perturbations were used during training. This method is essential to achieve occlusion invariance, a highly relevant property in clinical settings, as model performance deteriorates in the *occlusion test setting* without rich-representation learning. Furthermore, this approach does not introduce additional training parameters or computational complexity.

### 5.2. Landmark Refinement Methods

We explored explicit anatomical landmark refinement using the Graphical Model (GM), implicit anatomical constraints within the SpatialConfiguration-Net (SCN), and end-to-end trainable explicit anatomical constraints within our proposed Global Anatomical Feasibility Filter and Analysis (GAFFA) method. Furthermore, we utilized rich-representation learning with occlusions in all of these experiments, which represents another form of implicit anatomical constraint applied during training. In this way, we could demonstrate that explicit anatomical constraints are needed to enhance the robustness of ALL methods.

#### 5.2.1. Graphical Model

The GM, solved with Loopy Belief Propagation (LBP), greatly increases the robustness of U-Net predictions in our *occlusion test setting* by explicitly leveraging prior anatomical information. However, it also heavily increases the reported mean and median $PE_{all}$, especially in the *standard test setting*, where this landmark refinement method generally degrades initial localization performance for most metrics. We argue that this performance degradation can be partially attributed to the need for a blob peak extraction method to define landmark candidates, which introduces errors by assuming perfectly Gaussian blobs within a certain width range controlled by the σ parameter. Furthermore, this method is limited to selecting landmark candidates extracted from the corresponding prediction heatmap of a unique landmark and cannot introduce new candidates. Finally, the GM is a separate, non-differentiable component that cannot be integrated into end-to-end training.

Although graphical models have been effectively used for landmark refinement in previous studies [10,11], we argue that their performance largely depends on the uncertainty of shallow prediction methods, such as Regression Forests [10,41], which generate many landmark candidates for each unique landmark. In the context of deep-learning models trained on large datasets, which are confident in their predictions after convergence, our results show that the GM is severely outperformed by both the SCN and GAFFA across all metrics, except for Standard Deviation (SD) and large outliers, where it performs similarly to GAFFA. However, we argue that the use of the GM is justified in scenarios where models are uncertain (e.g., very small datasets) and provide sufficient landmark candidates such that there are anatomically feasible candidates to choose from for each unique landmark.

#### 5.2.2. SpatialConfiguration-Net

**Figure 10:**
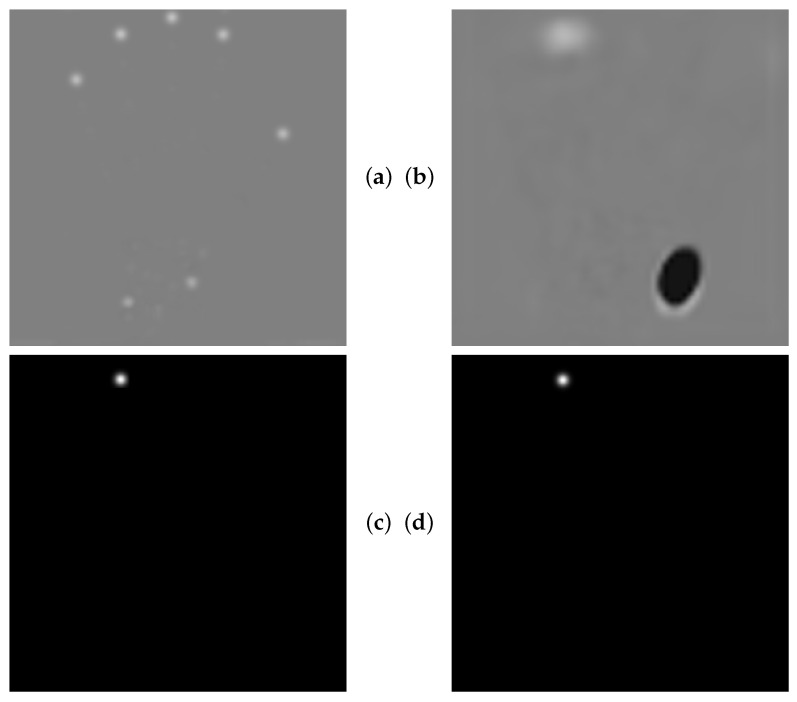
(**a**) Localization (U-Net) output. Locally accurate, but globally ambiguous landmark predictions. (**b**) Spatial configuration output. Spatial filter for landmark disambiguation. (**c**) Final SCN output. (**d**) Target heatmap. The gray areas in the top images are due to the fact that these images have a very different intensity distribution than the bottom images. Therefore, they are shown with different contrast settings to visually depict the intensity range within each image more clearly.

The SCN, in contrast to the Graphical Model, supports end-to-end training and relies on a weaker implicit anatomical constraint through its spatial configuration component. This component allows the overall model to partition the ALL task into localization and refinement during training. However, there is no guarantee that the model converges to this state, as the loss only evaluates the final heatmap computed with the Hadamard product between the localization prediction and the output of the spatial configuration component. Our experimental results deviate from those reported in Payer et al. [15], who showed that the SCN outperforms the U-Net. It can be argued that differences in our setup (e.g., use of the Adam optimizer, lower resolution, batch normalization in the spatial configuration component, and a batch size larger than one) could explain the different performance measurements compared to [15]. However, according to [15], the performance improvement of the SCN is mainly due to the addition of the spatial configuration component, which acts as a spatial filter and constrains the output to anatomical feasibility after convergence. After training the SCN in our framework, our localization component indeed converged to accurately report locally similar structures, and the spatial configuration component acted as a spatial filter, as illustrated in Figure 10.

Our results indicate that the anatomical constraint modeled within the SCN architecture is too weak for reliable outlier filtering. Additionally, the implicit partitioning of the ALL task into localization and spatial constraint in the SCN does not adapt to occlusion perturbations during training as effectively as using the U-Net alone. We hypothesize that this behavior is due to the constrained receptive field of the spatial configuration component, which is limited by its kernel size and cannot fully exploit information from neighboring landmark predictions that are not occluded when specific landmarks are blocked. Although the spatial configuration component uses very large kernels (resulting in a large number of learnable parameters) and operates in a highly downsampled image space, it does not undergo further downsampling during each convolution. Our results show that the U-Net can internally handle landmark disambiguation just as well without additional parameters. Similarly, GAFFA also has large kernels and operates in the same image space, but it includes a fixed internal mathematical model that ensures that occluded landmarks can be estimated by other landmark predictions with intact local appearance, while using prior anatomical information for kernel initialization.

#### 5.2.3. Global Anatomical Feasibility Filter and Analysis

GAFFA outperforms other landmark refinement methods in our framework across all experiments and test settings. This is achieved despite having a similar number of learnable parameters, operating in the same image space, using the same localization model training setup with occlusion perturbations, and the same training duration. In the *occlusion test setting*, GAFFA reduced the standard deviation to almost half the value compared to using the U-Net alone, while maintaining low mean and median $PE_{all}$ compared to the GM. Even using only the U-Net component trained with GAFFA showed more robustness to large outliers and lower SD in the *occlusion test setting* than the U-Net model trained alone. This demonstrates that GAFFA is a valuable component for end-to-end training with a U-Net, improving robustness to outliers after convergence, which is critical for ALL performance in clinical scenarios.

Our visualization results of GAFFA further confirm that this methodology is the most promising landmark refinement approach in our framework. The state of converged deep-learning models is often difficult to interpret, with no guarantee of robust handling of out-of-distribution inputs. GAFFA provides a fixed, differentiable mathematical constraint within a CNN that improves the robustness of landmark predictions by approximately solving a Markov Random Field (MRF) with a single iteration of the sum-product algorithm. This imposes a strong anatomical constraint on the predictions of the localization model by using pre-computed conditional distribution heatmaps that encode prior anatomical knowledge. In addition, this approach allows us to intuitively visualize the anatomical certainty of our approximated MRF solution by staying in image space. Staying in image space is a big advantage over the GM because it does not require inaccurate blob detection methods for landmark candidate extraction. Therefore, we found only a small increase in the mean and median point-to-point error compared to using the U-Net alone. Furthermore, GAFFA uses information from all prediction heatmaps to filter each unique landmark, where all pixels in our localization heatmap space are potential landmark candidates, which is another advantage over the Graphical Model.

Despite using grouped convolutions in Fourier space with FFT, a drawback of GAFFA is its inference time, which is almost six times longer than using the U-Net alone (GTX 1060 GPU and Intel i5 6600K CPU). This is due to the computationally expensive large convolution kernels used repeatedly in Equation (4). In addition, the definition of a dataset-specific graph topology, as shown in Figure 2, is necessary to handle occlusions without increasing the computational overhead, as each added global landmark is convolved with every other landmark in GAFFA.

Regarding related work, GAFFA could not outperform the reported performance of state-of-the-art approaches trained on much higher resolution inputs. However, it is unclear whether or not they used the same test setup with cross-validation as Payer et al. [15], as their test schemes are not described in detail. Only two of the papers [20,24] that outperform our model on the *standard test setting* mention three-fold cross-validation, but do not specify if they used the same splitting as [15]. Since they have not published their code, we cannot verify the correct splitting. For this reason, we have included a performance measurement of [3] for the studied hand dataset, which was taken from a results table in [20], as the original paper of [3] does not discuss experiments with this dataset.

However, we were able to outperform the SCN model of [15] on all metrics except mean and median $PE_{all}$, which are not as important as robustness to outliers in clinical practice, especially when localization serves as an initial step for more detailed segmentation or classification/regression tasks [33,34]. Furthermore, we outperformed a very recent state-of-the-art approach [17] on all metrics in the *standard test setting*, which also uses prior anatomical information during training and employs the coordinate regression scheme of DSNT. Importantly, the authors of [17] used the same test setup with the same cross-validation split as [15], and their code is publicly available. Finally, we argue that the reported performance of [22] is difficult to reproduce because they have not published any information about their test and training splits or their general testing scheme. They claim to have no outlier prediction more than 10 mm from the ground truth location. However, we argue that the found annotation error of the hand dataset makes it very difficult for converged models to report no outliers beyond 10 mm (our best model reports one 10 mm outlier). We further argue that models that agree with this annotation error are unlikely to understand the anatomical relationships as well as GAFFA and may have overfitted on the dataset. In addition, the explicit anatomical constraints within GAFFA are enforced during training and inference, which is not guaranteed by other methods in the literature that only use implicit anatomical constraints.

In cases of large outliers in the *occlusion test setting*, where strong occlusions can cause entire fingers to be missing, it becomes increasingly difficult to accurately predict the landmark location the closer the landmark is to the fingertips. In these cases, as shown in Figure 9, we cannot expect our model to be very accurate locally due to insufficient information; however, we are still able to provide a good estimate due to our incorporated explicit anatomical constraint.

Overall, these observations strongly suggest that, to the best of our knowledge, our approach is the most robust to large outliers in the literature at the time of writing, while still maintaining comparable performance on other metrics to other state-of-the-art methods.

## 6. Conclusions

Current ALL literature mainly focuses on implicit anatomical constraints to make ALL methods more robust against outlier predictions, which are detrimental for subsequent medical applications. This work investigates how well explicit anatomical constraints can improve the landmark refinement performance of ALL methods. We propose the end-to-end trainable Global Anatomical Feasibility Filter and Analysis (GAFFA) method that explicitly enforces anatomical constraints based on prior anatomical knowledge learned from the data. We compared the proposed method with two other landmark refinement techniques, the SpatialConfiguration-Net (SCN) and the Graphical Model (GM). SCN has an implicit anatomical constraint within the network, as it expects its two components to converge to a state where one acts as a locally accurate localizer, while the other is expected to act as a spatial filter. However, there is no guarantee that the model will converge to this state. The GM uses an explicit anatomical constraint by defining the landmark refinement task as a discrete optimization problem that is approximated in an external post-processing step. However, it cannot be closely integrated into an end-to-end trained model.

We tested all methods on the *standard test setting*, which we adopted directly from the literature, and the *occlusion test setting*, which brings the test setting closer to clinical practice, where occlusions may occur. The *occlusion test setting* demonstrated that the U-Net can capture the global relationship between anatomical structures without using attention, which is often thought to be necessary for CNNs to effectively learn global features [1,21]. By exposing it to artificial occlusions during training, the U-Net alone was able to accurately localize landmarks, even in the absence of local appearance information. However, despite using implicit anatomical constraints during training, it still requires the use of GAFFA to increase robustness against anatomically infeasible predictions.

We could demonstrate that GAFFA outperforms the other implemented landmark refinement paradigms GM and SCN in both test settings. Compared to the state-of-the-art methods, GAFFA shows a high level of robustness against large outliers while still performing well on other metrics. There are other methods [22] that claim to have zero outliers that are 10 mm away from the target position (we report one). However, we were able to show that there is an annotation error in the dataset, which prevents our model from reporting zero large outliers. This was possible due to visual inspection and the fact that we can visually analyze the learned anatomical constraint within GAFFA. Therefore, we argue that models that agree with this annotation error overfit on the dataset and cannot understand anatomy as well as GAFFA.

### Future Work

GAFFA may have shown high outlier robustness on this 2D dataset. However, we argue that current ALL methods are performance saturated on the studied, still widely used, hand dataset. Therefore, future work should focus on applying our proposed method to other datasets, e.g., [42], which are more complex and better represent occlusions that may occur in clinical practice. Furthermore, since GAFFA introduces computational overhead, it has to be investigated how to best extend our proposed method to 3D data. Moreover, we believe that there is potential for further automating the process of finding annotation errors with GAFFA that should be explored in future work. In addition, data efficiency is a very important property for models working on medical imaging tasks due to the sparsity of annotated data. Although we could not fully explore this aspect for our investigated models, preliminary experiments with only 50 training samples per fold indicated that GAFFA is more sensitive to weight initialization compared to other methods. Therefore, this aspect should also be further investigated in future experiments. Finally, to use GAFFA in a clinical setting, future work has to explore how to introduce a flag indicating whether a landmark is present (not occluded) in an image or not. This would allow physicians or subsequent medical applications to exclude the marked landmarks from further analysis.

## Figures and Tables

**Table 1:** Comparison of several models that were trained with and without occlusion on the *standard test setting*. Models were trained with heatmap regression, Mean Squared Error (MSE) loss and target heatmaps that contain Gaussian or Laplacian distributed blobs. Values highlighted in green mark the best result in their respective column. We average the performance over three fixed cross-validation folds per experiment.

Method	${\mathrm{PE}}_{\mathrm{all}}$ (in mm)			#O_r_ (%O_r_)		
	MD	μ	SD	r > 2 mm	r > 4 mm	r > 10 mm
U-Net Gaussian Blobs	**0.47**	**0.69**	±0.71	1787 (5.40%)	236 (0.71%)	**2 (0.006%)**
+ Occlusion Train	0.49	**0.69**	**±0.66**	**1534 (4.63%)**	**161 (0.49%)**	**2 (0.006%)**
U-Net Laplacian Blobs	**0.47**	**0.69**	±0.70	1678 (5.07%)	219 (0.66%)	**2 (0.006%)**
+ Occlusion Train	0.50	**0.69**	**±0.66**	**1540 (4.65%)**	**148 (0.45%)**	**2 (0.006%)**
SCN Gaussian Blobs	**0.48**	0.70	±0.74	1795 (5.42%)	260 (0.79%)	5 (0.015%)
+ Occlusion Train	0.49	**0.69**	**±0.66**	**1576 (4.76%)**	**152 (0.46%)**	**2 (0.006%)**

**Table 2:** Comparison between different landmark refinement methods on the *standard test setting*. All models were trained with occlusion perturbation and target heatmaps containing Gaussian-distributed blobs, unless otherwise noted. Models trained with Laplacian blobs are included for completeness, but they are not included in the final comparison. Bold values indicate the best and underlined values indicate the second best performance metric in that column.

Method	${\mathrm{PE}}_{\mathrm{all}}$ (in mm)			#O_r_ (%O_r_)		
	MD	μ	SD	r > 2 mm	r > 4 mm	r > 10 mm
U-Net	**0.49**	**0.69**	±0.66	**1534 (4.63%)**	161 (0.49%)	2 (0.006%)
U-Net+GM	0.67	0.83	±0.66	1790 (5.41%)	158 (0.48%)	2 (0.006%)
SCN	**0.49**	**0.69**	±0.66	1576 (4.76%)	152 (0.46%)	2 (0.006%)
**U-Net**+GAFFA	**0.49**	**0.69**	±0.66	1552 (4.69%)	158 (0.48%)	2 (0.006%)
U-Net+GAFFA	0.50	0.70	**±0.65**	1572 (4.75%)	**142 (0.43%)**	**1 (0.003%)**
**U-Net**+GAFFA Lapl. Blobs	0.49	0.69	±0.66	1525 (4.61%)	174 (0.53%)	2 (0.006%)
U-Net+GAFFA Lapl. Blobs	0.51	0.71	±0.65	1548 (4.67%)	143 (0.43%)	1 (0.003%)

**Table 3:** Comparison between different landmark refinement methods on the *occlusion test setting*. All models were trained with occlusion perturbation, except the models marked with WO. One model was trained with Laplacian blobs, but we do not highlight its performance because we want to compare landmark refinement methods as fairly as possible. Bold values indicate the best and underlined values indicate the second best performance metric in that column.

Method	${\mathrm{PE}}_{\mathrm{all}}$ (in mm)			#O_r_ (%O_r_)		
	MD	μ	SD	r > 2 mm	r > 4 mm	r > 10 mm
U-Net WO	0.52	3.80	±18.96	3935 (11.88%)	2275 (6.87%)	1442 (4.35%)
SCN WO	0.54	3.18	±11.08	4274 (12.90%)	2679 (8.09%)	2180 (6.58%)
U-Net	**0.53**	0.84	±2.03	2495 (7.53%)	562 (1.70%)	22 (0.07%)
U-Net+GM	0.70	0.95	**±1.06**	2767 (8.36%)	560 (1.69%)	**18 (0.05%)**
SCN	**0.53**	0.94	±2.56	2621 (7.91%)	736 (2.22%)	131 (0.40%)
**U-Net**+GAFFA	**0.53**	**0.83**	±1.54	**2482 (7.50%)**	581 (1.75%)	**18 (0.05%)**
U-Net+GAFFA	0.55	**0.83**	±1.14	2499 (7.55%)	**546 (1.65%)**	20 (0.06%)
**U-Net**+GAFFA Lapl. Blobs	0.53	0.81	±1.09	2465 (7.44%)	577 (1.74%)	15 (0.05%)
U-Net+GAFFA Lapl. Blobs	0.55	0.83	±1.00	2493 (7.53%)	512 (1.55%)	13 (0.04%)

**Table 4:** Comparison between our best model trained with three-fold cross-validation and state-of-the-art models on the *standard test setting*. Our model was trained with occlusion perturbations and we averaged the performance over three fixed cross-validation folds, the same as in [15,17]. The method marked with (*) is only mentioned for completeness, as we cannot verify if it used the same testing scenario as [15,17]. Other methods claim to have used three-fold cross-validation. Only a fraction of the methods in the literature report the absolute number of outliers. Therefore, we have estimated these values and marked them in italics. Other unknown values are marked with (?).

Method	${\mathrm{PE}}_{\mathrm{all}}$ (mm)			$\#O_{r}\;\left(\%O_{r}\right)$		
	MD	μ	SD	r > 2 mm	r > 4 mm	r > 10 mm
**1024 × 1216 input size**						
Viriyasaranon et al. [22] *	?	0.56	±0.58	? (3.17%)	? (0.37%)	? (0.0%)
**800 × 640 input size**						
Oh et al. [3] (from [20])	?	0.63	±0.71	*1301* (3.93%)	*109* (0.33%)	*3* (0.01%)
Kang et al. [24]	?	0.64	±0.64	*1311* (3.96%)	*112* (0.34%)	*6* (0.02%)
Ham et al. [20]	?	**0.61**	**±0.61**	***1231*** **(3.72%)**	***102*** **(0.31%)**	*3* (0.01%)
**512 × 512 input size**						
Payer et al. [15]	0.43	0.66	±0.74	1659 (5.01%)	241 (0.73%)	3 (0.01%)
Huang et al. [17]	?	0.72	±0.74	*1861* (5.62%)	*168* (0.51%)	*3* (0.01%)
**256 × 256 input size**						
U-Net+GAFFA (Ours)	0.50	0.70	±0.65	1572 (4.75%)	142 (0.43%)	**1 (0.003%)**

## Data Availability

The data presented in this study are available publicly. Upon acceptance, we will provide data and code for our test scenarios and models via a publicly available repository.

## Abbreviations

The following abbreviations are used in this manuscript:

ALL	Anatomical Landmark Localization
CPU	Central Processing Unit
GPU	Graphics Processing Unit
CNN	Convolutional Neural Network
SCN	SpatialConfiguration-Net
GM	Graphical Model
GAFFA	Global Anatomical Feasibility Filter and Analysis
MRF	Markov Random Field
GMM	Gaussian Mixture Model
LBP	Loopy Belief Propagation
FFT	Fast Fourier Transform
MSE	Mean Squared Error
DSNT	Differentiable Spatial to Numerical Transform
MD	Median
SD	Standard Deviation
WO	Without Artificial Occlusions
